# Spontaneous chromosomal instability in peripheral blood lymphocytes from two molecularly confirmed Italian patients with Hereditary Fibrosis Poikiloderma: insights into cancer predisposition

**DOI:** 10.1590/1678-4685-GMB-2020-0332

**Published:** 2021-08-06

**Authors:** Gaia Roversi, Elisa Adele Colombo, Ivana Magnani, Cristina Gervasini, Giuseppe Maggiore, Mauro Paradisi, Lidia Larizza

**Affiliations:** 1 University of Milano-Bicocca, School of Medicine and Surgery, Department of Medicine and Surgery, Monza, Italy.; 2 Università degli Studi di Milano, Genetica Medica, Dipartimento di Scienze della Salute, Milan, Italy.; 3 Bambino Gesù Children’s Hospital IRCCS, Division of Hepatology and Gastroenterology, Rome, Italy.; 4 Istituto Dermopatico dell’Immacolata, IDI-IRCCS, Laboratory of Molecular and Cell Biology, Rome, Italy.; 5 IRCCS Istituto Auxologico Italiano, Laboratorio di Citogenetica e Genetica Molecolare Umana, Milan, Italy.

**Keywords:** POIKTMP, *FAM111B* pathogenic variants, chromosomal instability, cancer predisposition, pancreatic carcinoma

## Abstract

Two Italian patients with the initial clinical diagnosis of Rothmund-Thomson syndrome were negative for *RECQL4* mutations but showed in peripheral blood cells a spontaneous chromosomal instability significantly higher than controls. Revisiting after time their clinical phenotype, the suggestive matching with the autosomal dominant syndrome Poikiloderma, Hereditary Fibrosing with Tendon Contracture, Myopathy and Pulmonary fibrosis (POIKTMP) was confirmed by identification of the c.1879A>G (p.Arg627Gly) alteration in *FAM111B*. We compare the overall clinical signs of our patients with those of reported carriers of the same mutation and present the up-to-date mutational repertoire of *FAM111B* and the related phenotypic spectrum. Our snapshot highlights the age-dependent clinical expressivity of POIKTMP and the need to follow-up patients to monitor the multi-tissue impairment caused by *FAM111B* alterations. We link our chromosomal instability data to the role of *FAM111B* in cancer predisposition, pointed out by its implication in DNA-repair pathways and the outcome of pancreatic cancer in 2 out of 17 adult POIKTMP patients. The chromosomal instability herein highlighted well connects POIKTMP to cancer-predisposing syndromes, such as Rothmund-Thomson which represents the first hereditary poikiloderma entering in differential diagnosis with POIKTMP.

Blood samples of two unrelated patients with a phenotype suggestive of a genodermatosis reminiscent of Rothmund-Thomson syndrome (RTS, MIM #268400) were sent to our laboratory in 2002 to perform *RECQL4* (MIM *603780) molecular analysis, after obtainment of informed consents. Briefly, the two Italian patients, a male (patient A) and a female (patient B) both representing the only child born to non-consanguineous parents, presented typical RTS clinical signs, such as poikiloderma, sparse and rare hair, eyelashes and eyebrows, short stature, and photosensitivity, combined with peculiar features, mainly oedema, muscular weakness and liver dysfunction with vanishing bile duct syndrome since infancy, rarely or never reported in RTS. Their clinical conditions deteriorated with time and the liver involvement in patient A caused ascites and sepsis that led him to premature death at age 17 years. Patient B also had a liver impairment: she suffered from idiopathic inflammatory cholangiopathy with ductopenia, as attested by hepatic biopsy, but had a much less impaired liver function; unfortunately, she prematurely died following a trauma that occurred when she was 15 years old. Sanger sequencing of the entire *RECQL4* gene (NG_016430.1), 21 exons and 20 introns, except for IVS12 minisatellite (Colombo *et al*., 2018), was carried out but did not detect any pathogenic alteration.

In parallel to *RECQL4* gene scan, spontaneous and induced chromosomal instability, a cellular hallmark of RTS syndrome (Larizza *et al*., 2010) and other autosomal recessive syndromes driven by defects in *RECQ* helicase genes (such as Werner Syndrome, MIM #277700) or entering in differential diagnosis with RTS (such as Fanconi Anemia, MIM Phenotypic Series-PS227650) was monitored on heparinized peripheral blood lymphocytes from the two probands and six healthy controls, including both probands’ parents. A minimum of 50 metaphase spreads prepared according to standard procedures and QFQ-banded were analysed per sample. As shown in Table 1, a significantly higher number of chromatid and chromosome gaps and breaks per metaphase was highlighted in both probands (patient A: 0.25 (13/52), patient B: 0.22 (11/51)) as compared to controls (0.11 (34/318)) (p<.05 at the chi-square test). The chromosomal lesions, widespread in the genome, involved different chromosomal locations, rarely coincident with fragile sites, as apparent in the set of eight different Q-banded chromosomes (Figure 1). Conversely, the search for chromosomal fragility after aphidicolin treatment (0.4 M for 24 hr) did not evidence a rate of chromosome lesions significantly higher in the probands than in parallel control lymphocytes cultures as the ratio of aberrations/metaphases was 3.21 in patient A, 4.96 in patient B, and 4.52 in controls.

**Table 1:** Spontaneous chromosomal instability in peripheral blood cells from two unrelated patients with a Rothmund-Thomson-like phenotype.

	Patients		Controls						
Observed aberrations	Patient A	Patient B	Controls total	Parents of patient A		Parents of patient B		Control 1	Control 2
				Mother	Father	Mother	Father		
Chromatid Gaps	7	4	19	1	2	5	3	5	3
Chromatid Breaks	1	0	5	1	0	3	0	0	1
Chromosome Gaps	1	4	6	1	0	2	0	2	1
Chromosome Breaks	4	3	4	1	1	1	1	0	0
Total aberrations	13	11	34	4	3	11	4	7	5
No. scored metaphases	52	51	318	50	54	53	56	54	51
Aberrations/ metaphase	0.25*	0.22*	0.11*	0.08	0.06	0.21	0.07	0.13	0.10

* Significant at p < .05 at the chi-square test

**Figure 1 -:**
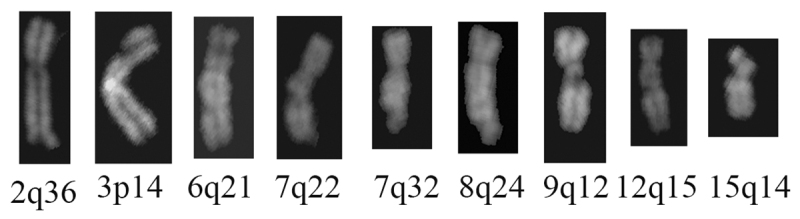
Spontaneous breaks and gaps in two unrelated Italian patients with a clinical presentation reminiscent of Rothmund-Thomson syndrome. Selected representative images document the spontaneous breaks and gaps observed in the QFQ-banded chromosome spreads from blood samples of the two patients herein reported.

As the molecular identity of the syndromic condition of the two patients was unknown at that time this data could neither be associated with a specific rare syndrome nor corroborated by testing similarly affected patients.

Many years later, the phenotype of our probands was revisited in the context of the literature. We noted a reasonable match between the phenotype of our cases and that of patients with the ultra-rare autosomal dominant syndrome Poikiloderma, Hereditary Fibrosing, with Tendon contracture, Myopathy and Pulmonary fibrosis (POIKTMP, MIM #615704) (Khumalo *et al*., 2006; Mercier *et al*., 2013). Hence, targeted Sanger sequencing of the POIKTMP-causative gene *FAM111B* (MIM #615584), encoding a trypsin-like cysteine/serine peptidase (Mercier *et al.,* 2013), highlighted on both our patients the monoallelic c.1879A>G alteration (NG_034129.1) (Figure 2A). This pathogenic variant is not present in the DNA of patient B’ mother, the only parent we could test. Moreover, we detected this alteration in the probands’ lymphoblastoid cell lines RNA (data not shown) confirming the stability of the mutated *FAM111B* transcript r.1879A>G (p.Arg627Gly).

**Figure 2 -:**
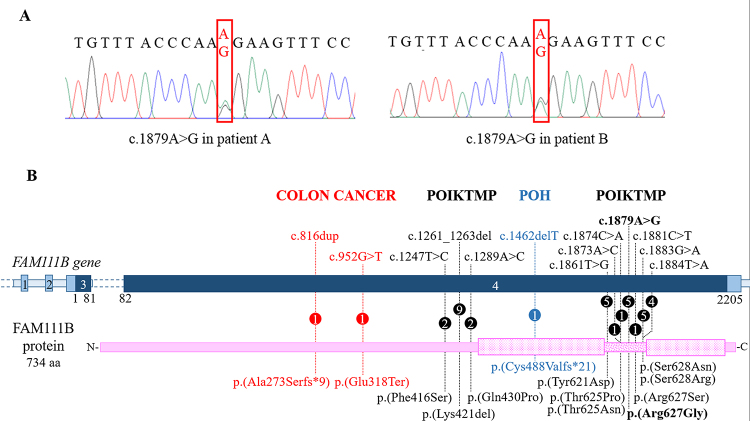
The mutational repertoire of *FAM111B*. A) Electropherograms showing the c.1879A>G *FAM111B* missense variant (framed by the red rectangle) detected in the two unrelated Italian patients. B) Picture summarizing the *FAM111B* mutational repertoire. The dark blue bar indicates the coding regions of the gene while the non-coding regions are in light blue. The pink bar indicates the protein: the punctate boxes correspond to the protease domain, spanning from amino acid residues 471 to 664 and interrupted by a linker region (dotted bar). Each change is described at DNA (top) and protein level (below). Numbers within the circles specify the number of patients carrying the variant. Pathogenic variants associated with POIKTMP, POH, and colon cancer are written in black, blue and red, respectively.

The identified missense pathogenic variant affects a highly conserved amino acid residue placed in the loop that splits into two parts the functional enzymatic domain of FAM111B (Mercier *et al*., 2013) (Figure 2B). Of note, six additional *FAM111B* mutations map in this linker region (41 amino acids in length) that can be considered the major mutation cluster; a minor cluster of three pathogenic variants maps upstream the catalytic domain (Figure 2B).

Including our cases, the c.1879A>G (p.Arg627Gly) mutation has been reported so far in five patients from four families (Figure 2B and Table 2): our two from Italy, namely patient A and patient B (alias F3-II1 reported by Mercier *et al*., 2013, 2015), a third one from France (patient C, F1-II2, Mercier *et al.,* 2013, 2015), and two from Algeria (patient D, F2-II4, Mercier *et al.,* 2013, 2015, 2019, and his son patient E, F2-III1, Mercier *et al.,* 2015). Table 2 summarizes the clinical signs of the reported POIKTMP cases sharing the hot spot c.1879A>G (p.Arg627Gly) pathogenic variant. A detailed clinical report of patient B marking the evolution of the disease can be found in Supplementary File 1.

**Table 2 -:** Summary of the clinical signs observed in patients with the c.1879A>G (p.(Arg627Gly)) *FAM111B* mutation.

	Patient A	Patient B/F3-II1^a,b^	Patient C/F1-II2^a,b^	Patient D/F2-II4^a,b,c^	Patient E/F2 son^b^
Origin	Italy	Italy	France	Algeria	Algeria
Age at clinical evaluation	2y	4y	7y	30y	8m
Sex	Male	Female	Male	Male	Male
Short stature	+ (3° percentile)	+ (50° percentile)	-	+	-
Skin					
Poikiloderma	+	+	+	+	+
Cellulitis	-	+	+	-	-
Dermatitis	+	+	-	-	-
Eczematous lesions	-	-	+	+	+
Sclerosis of digits	-	-	-	+	-
Vitiligo	-	+	-	-	-
Sparse scalp hair	+	+	+	+	+
Sparse eyelashes	+	+	+	+	+
Sparse eyebrows	+	+	+	+	+
Heat intolerance	+	+	+	+	n.d.
Eyes Cataract	-	+	-	-	-
Pancreas					
Insufficiency	-	-	+	-	n.d.
Cancer	-	-	-	+	n.d.
Liver					
Failure	+	-	n.d.	-	-
Vanishing bile ducts	+	+	n.d.	-	-
Cirrhosis	+	-	n.d.	-	-
Cholestasis	+	+	n.d.	-	-
Hepatomegaly	-	+	n.d.	-	
Lungs					
Asthma	-	-	+	-	n.d.
Restrictive syndrome	-	-	-	+	n.d.
Muscle					
Weakness	+	+	+	+	-
Atrophy	-	-	+	+	-
Adiposis	-	+	+	+	-
Contractures	+	+	+	+	-
Lymphedema	+	+	+	-	-
Haematological alterations	↓ IgG1, IgG2, IgG4, ↑ IgE ↑ GoT ↑ GPT ↑ gammaGT	↓ IgG2, IgG4, ↑ IgG1 ↑ GoT ↑ GPT ↑ gammaGT ↑ LDH ↑ CPK	Eosinophilia	↑ CPK	n.d.
Others	Death at 17y for liver cirrhosis	Scoliosis Nail dysplasia Death at 15y for injury	n.d.	Scoliosis Dysphagia Death at 32y for cancer	n.d.

n.d.: not documented; a: Mercier *et al*., 2013; b: Mercier *et al.*, 2015; c: Mercier *et al.*, 2019

Briefly, all patients showed in the first year of life poikiloderma, predominant in the sun-exposed skin areas, and hypotrichosis involving hair, eyelashes, and eyebrows. Then, except patient E who was in the first childhood when the disease is not yet fully expressed, all manifested muscle weakness and limb contractures starting from childhood and/or adolescence. Patient C (F1-II2) suffered from asthma since infancy, while the adult patient D (F2-II4) developed a severe and abnormal lung function (Mercier *et al*., 2013, 2015). Both the Italian patients showed vanishing bile duct syndrome since infancy and liver impairment. Transaminitis and bile duct loss, not associated with cholestasis, were also detected in a patient with the c.1881C>T mutation affecting the same amino acid residue [p.(Arg627Ser)] (Dokic *et al*., 2020). Three patients prematurely died: patient A died as above mentioned for decompensated liver cirrhosis at age 17y; patient B had an accidental death at 15y, and patient D, the eldest carrier of c.1879A>G mutation, died at 32y due to intraductal pancreatic adenocarcinoma (Mercier *et al.,* 2019).

The pancreatic dysfunction is considered a relatively frequent clinical findings of POIKTMP: it has been diagnosed in five patients (8, 10, 28, and 58-years old) from a multiplex family co-segregating with the in-frame p.(Lys421del) alteration (Seo *et al*., 2016). Moreover, a 64-years-old patient with p.(Ser628Arg) mutation died for pancreatic carcinoma pointing to two cases with this rare cancer in the cohort of 17 adult POIKTMP patients who could be followed-up (Goussot *et al*., 2017; Mercier *et al*., 2019). Another POIKTMP clinical sign responsible for severe evolution of the disease is pulmonary fibrosis causing respiratory failure in three patients carrying mutations within both the major (p.(Arg627Gly); p.(Thr625Asn)) (Mercier *et al.*, 2013, 2015) or the minor (p.(Gln430Pro)) (Mercier *et al.*, 2015; Sanchis-Borja *et al*., 2018) mutation clusters (Figure 2B). The position of the mutation per se may not have a great influence on the phenotype as exemplified by the patient carrying the p.(Gln430Pro) mutation deceased for respiratory failure (Sanchis-Borja *et al.,* 2018) and the lack of restrictive pulmonary disease in a 14-years old girl carrying the same variant (Takeichi *et al*., 2017). Age-dependent clinical expressivity concurs to the phenotype as the multi-tissue/organs impairment caused by *FAM111B* mutation becomes increasingly manifest with age.

Comprehension of the genotype-phenotype relationship is made further trickier by the identification of the first frameshift *FAM111B* c.1462delT (p.(Cys488Valfs*21)) variant (Figure 2B), subverting the entire cysteine/serine peptidase domain, in a boy with Progressive Osseous Heteroplasia (POH), who also displayed classic POIKTMP signs such as tendon contractures, muscle weakness and ulcerated erupted skin lesions (Panjawatanan *et al*., 2019).

Furthermore, *FAM111B* germline variants have been also detected in two patients with colorectal cancer in the context of a genomic study searching for predisposing variants in genes involved in homologous recombination repair associated with hereditary cancer (Bertelsen *et al*., 2019). Interestingly the two loss-of-function germline alterations, c.816dup (p.(Ala273Serfs*9)) and c.952G>T (p.Glu318Ter) (Figure 2B), predicting early protein truncation/mRNA degradation, are not associated with a POIKTMP phenotype, raising the possibility that POIKTMP, POH, and non-syndromic cancer predisposition are allelic disorders driven by different types of *FAM111B* mutation.

The assumption that *FAM111B* pathogenic variants associated with POIKTMP act by gain-of-function or dominant-negative effect (Mercier *et al*., 2013) has been recently supported by the discovery that *FAM111B* mutations exacerbate the catalytic activity repressing DNA replication and transcription and triggering cell death (Hoffmann *et al*., 2020).

Several data extrapolated from the literature support this hypothesis. An indirect role of *FAM111B* in the NER and NHEJ repair pathways is inferred by its synthetic lethality with MLN4924, a small molecule of the NEDD8-activating enzyme inhibitor used in clinical trials for solid tumours and haematological malignancies (Blank *et al*., 2013). Moreover, FAM111B interacts with CAPNS1 (https://www.genecards.org/cgi-bin/carddisp.pl?gene=CAPNS1), a regulator subunit of m-calpain and µ-calpain, the two ubiquitously best-characterized proteases belonging to the calpain family. Interestingly Mercier and collaborators showed a decreased quantity of calpain on immunoblotted protein lysates from the muscle of patient D (F2-II4) (Mercier *et al.,* 2013). A link with DNA-repair pathways may be grasped as the CAPNS1-CAPN1 complex is involved in the stability of the USP1 deubiquitinase that modifies both FANCD2, a central node in different DNA damage response pathways (Lopez-Martinez *et al*., 2016), and PCNA involved in DNA metabolism (Choe and Moldovan, 2017).

Furthermore, transcriptomic findings of pancreatic cells treated for 48 h with metformin and aspirin had included *FAM111B* among the 10 top downregulated genes mainly involved in G1/S checkpoint regulation (Yue *et al*., 2015). Merging observations on a high *FAM111B* expression during the S phase suggest the protein may play a role in DNA replication (Aviner *et al*., 2015). Last, *FAM111B* has been implicated in susceptibility to prostate cancer by a genome-wide association study (Akamatsu *et al*., 2012) and it has been indicated as an unfavourable prognostic marker in pancreatic and liver cancer (https://www.proteinatlas.org/ENSG00000189057-FAM111B).

Our data evidenced an increased frequency of spontaneous chromosome gaps and breaks in peripheral blood lymphocytes from our two *FAM111B*-mutated patients as compared to healthy controls. Values of normal controls also appear higher than those reported in other case studies of the literature but they may be explained by physiological issues (including lifestyle) or linked to an overestimate of aberrations due to the evaluation of QFQ-banded chromosomes, instead of Giemsa stained chromosomes, usually preferred to detect gaps and breaks.

By linking our data to the above evidence, we speculate that detection of increased chromosome gaps and breaks in peripheral blood lymphocytes from 2 *FAM111B*-mutated patients could reflect (secondary manifestation) an impairment of one of the DNA replication/DNA-damage repair pathways (primary defects).

Though mechanistic insights on *FAM111B* role remain to be elucidated and integrated, they are consistent with the increased spontaneous instability noted in *FAM111B* mutated cells. It could be envisaged that stem/progenitor cells of pancreatic ductal epithelia are especially sensitive to the effect of *FAM111B* mutations. Further POIKTMP patients and cell types should be analysed to confirm and expand our data. As the instability assay was performed on patients in early childhood, should it be significant in additional POIKTMP patients, it may be used as an adjunct test for diagnosis and oncological surveillance.

The herein highlighted chromosomal instability represents a hallmark that definitely links POIKTMP to cancer-predisposing syndromes driven by disruption of the mechanisms safeguarding genomic stability (Taylor *et al*., 2019), particularly Rothmund-Thomson type II syndrome where patients cells show centromere mis-division (Larizza *et al*., 2006) and fragile telomeric ends (Ghosh *et al*., 2012), and Dyskeratosis Congenita (DK) characterized by short telomeres (Savage, 2018). It is thus not surprising that POIKTMP, RTS-II, and DK display a few overlapping features, such as growth delay and sparse hair, pigmentation changes, and cancer predisposition. POIKTMP and RTS-II patients also share poikiloderma, sparse eyelashes/eyebrows, and the less common sign of sclerosis of the digits, while both POIKTMP and DK patients are prone to pulmonary fibrosis and liver disease. The diagram in Figure S1 illustrates these clinical commonalities, together with the more numerous specific features of POIKTMP, RTS-II, and DK, which should be carefully considered for their differential diagnosis.

## Supplementary material

The following online material is available for this article:

Supplementary File 1 - Clinical report about the evolution of the disease in patient B.

Figure S1 - Common and specific clinical features of POIKTMP, RTS-II, and DK.

## References

[B1] Akamatsu S, Takata R, Haiman CA, Takahashi A, Inoue T, Kubo M, Furihata M, Kamatani N, Inazawa J, Chen GK (2012). Common variants at 11q12, 10q26 and 3p11.2 are associated with
prostate cancer susceptibility in Japanese. Nat Genet.

[B2] Aviner R, Shenoy A, Elroy-Stein O, Geiger T (2015). Uncovering hidden layers of cell cycle regulation through
integrative Multi-omic analysis. PLoS Genet.

[B3] Bertelsen B, Tuxen IV, Yde CW, Gabrielaite M, Torp MH, Kinalis S, Oestrup O, Rohrberg K, Spangaard I, Santoni-Rugiu E (2019). High frequency of pathogenic germline variants within homologous
recombination repair in patients with advanced cancer. NPJ Genom Med.

[B4] Blank JL, Liu XJ, Cosmopoulos K, Bouck DC, Garcia K, Bernard H, Tayber O, Hather G, Liu R, Narayanan U (2013). Novel DNA damage checkpoints mediating cell death induced by the
NEDD8-activating enzyme inhibitor MLN4924. Cancer Res.

[B5] Choe KN, Moldovan GL (2017). Forging ahead through darkness: PCNA, still the principal
conductor at the replication fork. Mol Cell.

[B6] Colombo EA, Locatelli A, Cubells Sánchez L, Romeo S, Elcioglu NH, Maystadt I, Esteve Martínez A, Sironi A, Fontana L, Finelli P (2018). Rothmund-Thomson Syndrome: Insights from New Patients on the
Genetic Variability Underpinning Clinical Presentation and Cancer
Outcome. Int J Mol Sci.

[B7] Dokic Y, Albahrani Y, Phung T, Patel K, de Guzman M, Hertel P, Hunt R (2020). Hereditary fibrosing poikiloderma with tendon contractures,
myopathy, and pulmonary fibrosis: Hepatic disease in a child with a novel
pathogenic variant of FAM111B. JAAD Case Rep.

[B8] Ghosh AK, Rossi ML, Singh DK, Dunn C, Ramamoorthy M, Croteau DL, Liu Y, Bohr VA (2012). RECQL4, the protein mutated in Rothmund-Thomson syndrome,
functions in telomere maintenance. J Biol Chem.

[B9] Goussot R, Prasad M, Stoetzel C, Lenormand C, Dollfus H, Lipsker D (2017). Expanding phenotype of hereditary fibrosing poikiloderma with
tendon contractures, myopathy, and pulmonary fibrosis caused by FAM111B
mutations: Report of an additional family raising the question of cancer
predisposition and a short review of early-onset
poikiloderma. JAAD Case Rep.

[B10] Hoffmann S, Pentakota S, Mund A, Haahr P, Coscia F, Gallo M, Mann M, Taylor NM, Mailand N (2020). FAM111 protease activity undermines cellular fitness and is
amplified by gain-of-function mutations in human disease. EMBO Rep.

[B11] Khumalo NP, Pillay K, Beighton P, Wainwright H, Walker B, Saxe N, Mayosi BM, Bateman ED (2006). Poikiloderma, tendon contracture and pulmonary fibrosis: a new
autosomal dominant syndrome?. Br J Dermatol.

[B12] Larizza L, Magnani I, Roversi G (2006). Rothmund-Thomson syndrome and RECQL4 defect: splitting and
lumping. Cancer Lett.

[B13] Larizza L, Roversi G, Volpi L (2010). Rothmund-Thomson syndrome. Orphanet J Rare Dis.

[B14] Lopez-Martinez D, Liang CC, Cohn MA (2016). Cellular response to DNA interstrand crosslinks: the Fanconi
anemia pathway. Cell Mol Life Sci.

[B15] Mercier S, Küry S, Shaboodien G, Houniet DT, Khumalo NP, Bou-Hanna C, Bodak N, Cormier-Daire V, David A, Faivre L (2013). Mutations in FAM111B cause hereditary fibrosing poikiloderma with
tendon contracture, myopathy, and pulmonary fibrosis. Am J Hum Genet.

[B16] Mercier S, Küry S, Salort-Campana E, Magot A, Agbim U, Besnard T, Bodak N, Bou-Hanna C, Bréhéret F, Brunelle P (2015). Expanding the clinical spectrum of hereditary fibrosing
poikiloderma with tendon contractures, myopathy and pulmonary fibrosis due
to FAM111B mutations. Orphanet J Rare Dis.

[B17] Mercier S, Küry S, Nahon S, Salort-Campana E, Barbarot S, Bézieau S (2019). FAM111B mutation is associated with pancreatic cancer
predisposition. Pancreas.

[B18] Panjawatanan P, Ryabets-Lienhard A, Bakhach M, Pitukcheewanont P (2019). MON-512 a de novo frameshift mutation of FAM111B gene resulting
in progressiveosseous heteroplsia in an African American boy: First case
report. J Endocr Soc.

[B19] Sanchis-Borja M, Pastré J, Mercier S, Juvin K, Benattia A, Israël-Biet D (2018). Pulmonary fibrosis associated with hereditary fibrosing
poikiloderma caused by FAM111B mutation: A case report. Rev Mal Respir.

[B20] Savage SA (2018). Beginning at the ends: telomeres and human
disease. F1000Res.

[B21] Seo A, Walsh T, Lee MK, Ho PA, Hsu EK, Sidbury R, King MC, Shimamura A (2016). FAM111B mutation is associated with inherited exocrine pancreas
dysfunction. Pancreas.

[B22] Takeichi T, Nanda A, Yang HS, Hsu CK, Lee JY, Al-Ajmi H, Akiyama M, Simpson MA, McGrath JA (2017). Syndromic inherited poikiloderma due to a de novo mutation in
FAM111B. Br J Dermatol.

[B23] Taylor AMR, Rothblum-Oviatt C, Ellis NA, Hickson ID, Meyer S, Crawford TO, Smogorzewska A, Pietrucha B, Weemaes C, Stewart GS (2019). Chromosome instability syndromes. Nat Rev Dis Primers.

[B24] Yue W, Wang T, Zachariah E, Lin Y, Yang CS, Xu Q, DiPaola RS, Tan XL (2015). Transcriptomic analysis of pancreatic cancer cells in response to
metformin and aspirin: an implication of synergy. Sci Rep.

