# Supplementary material for: Spontaneous chromosomal instability in peripheral blood lymphocytes from two molecularly confirmed Italian patients with Hereditary Fibrosis Poikiloderma: insights into cancer predisposition
Source: Genet Mol Biol. 2021 Aug 6;44(3):e20200332. doi: 10.1590/1678-4685-GMB-2020-0332 (PMC8345126; doi:10.1590/1678-4685-GMB-2020-0332)
Supplement: Supplementary File 1 - [file 1415-4757-GMB-44-3-e20200332-s1.pdf]

Genetics and Molecular Biology

https://doi.org/ 10.1590/1678-4685-GMB-2020-0332

Supplementary Material to "Spontaneous chromosomal instability in peripheral blood lymphocytes from two molecularly confirmed Italian patients with Hereditary Fibrosis Poikiloderma: insights into cancer predisposition"

Supplementary File 1 - Clinical report about the evolution of the disease in patient B.

Patient B was born at term by eutocic delivery with a birth weight of 2750 gr; she presented a fracture of the right clavicle and persistent neonatal jaundice.

At three months poikiloderma appeared on the face, neck, and upper limbs.

At five months the ALT levels were 4x, CPK 1,5x, IgG levels were low (184mg/dl).

At 8 months, weight was at 50-75th percentile, length 97th percentile. Brown palmoplantar erythema was noted. She had hepatomegaly with liver ultrasound within normal parameters, but biochemical indices of liver dysfunction and cholestasis (ALT 7.7x, AST 5.6x, GGT 6x, alkaline phosphatase 1.2x, bile acids 8.4 mcmol/L). Hypogammaglobulinemia (gamma globulins 0,3 g/dl, IgG 231 mg/dl) was recorded, with negative autoimmune serology for mitochondrial (AMA), anti-Liver–Kidney Microsomal (LKM), anti-Smooth Muscle (SMA), antinuclear (ANA) antibodies. Hypertriglyceridemia and increased CPK (306 U/L) were present. The clinical conclusion was intrahepatic chronic cholestasis, asymptomatic hypogammaglobulinemia, and suspected vitiligo.

At 12 months, the areas of leukoderma involved the abdomen and inguinal/pubic regions. Alopecia aerata involving the scalp, eyelashes, and eyebrows was apparent.

Face skin biopsy showed epidermis of normal thickness without inflammation of the reticular and papillary dermis. Lack of melanocytes. Biopsy of the skin of the leg evidenced orthokeratotic hyperkeratosis with hypergranulosis and Ectasia with acanthosis, ectasia of capillaries in the papillary dermis, oedema, lymphoid and histiocytic infiltrate, and accumulation of melanin in macrophages.

Liver biopsy showed inflammation with a mixed infiltration of polynucleated cells and lymphocytes, pericholangitis, porto-portal bridges, and progressive bile duct loss. This finding and the persistence of cholestasis and liver disfunction led to the diagnosis of "idiopathic inflammatory cholangiopathy with ductopenia", treated with ursodeoxycholic acid (UDCA) with good results.

At 3 years and 4 months leukoderma involved most of the skin surface as lenticular areas. Moreover, hyperpigmented, atrophic, and erythematous lesions appeared, besides to telangiectasia, especially on light-exposed skin areas. Oedema of the hands appeared.

The onset of asthenia and difficulties in walking, the persistence of increased CPK values (281 U/L) and clinical evaluations, which detected a mild hypotrophy/hypotonia of the shoulder girdle and the quadriceps muscles, and lumbar hyperlordosis, raised the suspicion of a myopathy. Muscle biopsy turned out to be of no diagnostic utility. Electromyography evidenced muscle sufferance. Conduction speed was in the normal range. An ophthalmologic check evidenced a polar point lens opacity of the right eye and small peripheral lens opacity of the left eye not compromising the visual acuity.

Biochemical analyses showed AST 4x, ALT 4x, GGT 3x, increased LDH, iron deficiency, hypochromic microcytic anaemia, IgG2 and IgG4 deficit. Negative the search for autoimmune antibodies (AMA, SMA, ANA, anti-SCL70, J01). The hepatologist confirmed the diagnosis of vanishing bile duct syndrome.

At 5 years and 4 months, hepatic transaminases and GGT were within the normal range, with increased bile acids (54 mcmol/L) and alkaline phosphatase (950 U/L). No more hepatosplenomegaly. Weight was at 3rd and height at 60th percentile. General conditions were fair. The girl was not able to run and showed a steppage gait, hypotonia and hypotrophy. Lymphedema of the lower right limb and foot was present as well as foot with internal rotation and supination on the march, due to muscle imbalance.

The orthopaedic evaluation revealed a right stiff clubfoot to the right and an early left clubfoot, probably of neuromuscular origin. Surgery for stretching of the sural triceps and posterior tibial was taken into account. After a few months, general conditions became poor.
