# Supplementary material for: Spontaneous chromosomal instability in peripheral blood lymphocytes from two molecularly confirmed Italian patients with Hereditary Fibrosis Poikiloderma: insights into cancer predisposition
Source: Genet Mol Biol. 2021 Aug 6;44(3):e20200332. doi: 10.1590/1678-4685-GMB-2020-0332 (PMC8345126; doi:10.1590/1678-4685-GMB-2020-0332)
Supplement: Figure S1 - [file 1415-4757-GMB-44-3-e20200332-s2.pdf]

Genetics and Molecular Biology

https://doi.org/ 10.1590/1678-4685-GMB-2020-0332

Supplementary Material to "Spontaneous chromosomal instability in peripheral blood lymphocytes from two molecularly confirmed Italian patients with Hereditary Fibrosis Poikiloderma: insights into cancer predisposition"

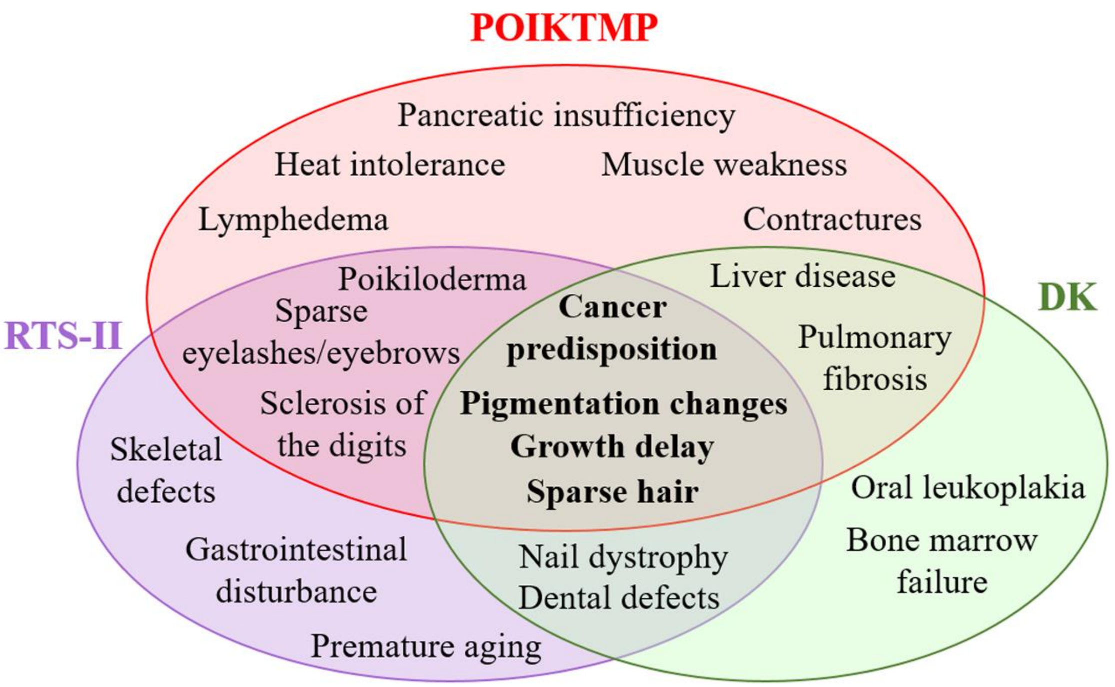

Figure S1 - Common and specific clinical features of POIKTMP, RTS-II, and DK.

Diagram illustrating the main clinical signs (black-bolded characters) shared by patients with the three syndromes having POIKTMP as reference. Distinctive features of each disorder are indicated in red for POIKTMP, violet for RTS-II, and green for DK.
